# Gravi-Sensitivity of Mosses and Their Gravity-Dependent Ontogenetic Adaptations

**DOI:** 10.3390/life12111782

**Published:** 2022-11-04

**Authors:** Oksana V. Lobachevska, Natalia Y. Kyyak, Elizabeth L. Kordyum, Yaroslava D. Khorkavtsiv, Volker D. Kern

**Affiliations:** 1 Institute of Ecology of the Carpathians, National Academy of Sciences of Ukraine, Kozelnytska Str. 4, 79005 Lviv, Ukraine; 2 M.G. Kholodny Institute of Botany, National Academy of Sciences of Ukraine, Tereschenkivska Str. 2, 01601 Kyiv, Ukraine; 3 Paragon Space Development Corporation, 3400 East Britannia Drive, Tucson, AZ 85706, USA

**Keywords:** microgravity, clinorotation, gravi-sensitivity, gravi-morphoses, mosses, protonemata, ontogenesis, adaptation

## Abstract

Gravi-morphoses affect the variability of plants and are the morphogenetic adaptation to different environmental conditions. Gravity-dependent phenotypic plasticity of gametophytes as well as gravi-sensitivity of moss protonemata in microgravity and simulated microgravity conditions are discussed. The moss protonema, a filamentous multicellular system, representing a juvenile stage of moss development, develops as a result of the elongation and division of the apical cell. This apical cell of the protonema is a unique object for research on moss gravi-sensitivity, as graviperception and gravitropic growth occur within the same single cell. Attention is focused on the influence of gravity on bryophyte ontogenesis, including the gravitropic reactivity of moss protonemata, gravi-sensitivity at the stage of leafy shoot development and sporogonium formation, gravity-influenced morphogenesis of apical cell budding, and gravity-dependent spiral growth patterns. The role of gravireceptors in the growth processes of mosses at the cellular level under microgravity conditions are being discussed, as well as the involvement of auxin transport, Ca2+-induced gravitropism and the cytoskeleton in gravitropic reactions.

## 1. Introduction

Gravity is a constant environmental factor that influences the evolutionary development of life on Earth. A fundamental uniqueness of plants is the high plasticity of their growth as a reaction to external influences. Tropisms are growth-mediated plant movements that allow plants to respond to changes in their environment. Gravitropism is the response to sensing the constant 1 g stimulus; gravity sensing involves the sedimentation of dense amyloplasts within specialized gravity-sensing cells, e.g., root and shoot statocytes. Gravitropism has been observed and analyzed in a variety of plant organs, including roots, hypocotyls, and inflorescence stems. Single, initially apolar cells, such as the *Fucus* zygote, spores, pollen grains, protonema of mosses and *Chara* algae serve as models to investigate the underlying mechanisms of gravitropism [1,2,3]. The perception of the gravity signal plays a key role in plant development: from the growth orientation of seedlings to the development of adult plants [4,5,6]. Positive gravitropic growth of the main root as well as the gravitropic reaction of lateral roots determine the plant’s overall structural architecture [7,8,9]. In the evolutionary chain, mosses belong to the oldest/earliest group of terrestrial plants. The unique ability of moss protonemata, in particular their apical cell, to both perceive and respond to the gravi-stimulus makes mosses as a model system for this type of research [10,11,12,13].

The spore, the reproductive unit that ultimately develops into an adult plant, is usually one-celled. When spores of mosses germinate in the light, they initially form a sporeling (chloronema and rhizoid), followed by juvenile filamentous structures referred to as protonemata and, subsequently, the adult gametophore forms. The chloronema, the primary photosynthetic stage of the protonema, is characterized by perpendicular cross walls, short cells, numerous chloroplasts, colorless cell walls, and irregular branching. Dendroids consist of tree-like branched chloronemata. Caulonemata, the secondary stage of protonema development, give rise to buds and upright gametophores. Caulonemata are characterized by longer cells with slanting cross walls and usually brownish cell walls. In mosses, rhizoids, the anchoring and absorbing structures, are multicellular featuring oblique end walls. The moss sporangium (the seta and capsule), which produces spores, is the main body of the sporophyte.

**Figure 1:**
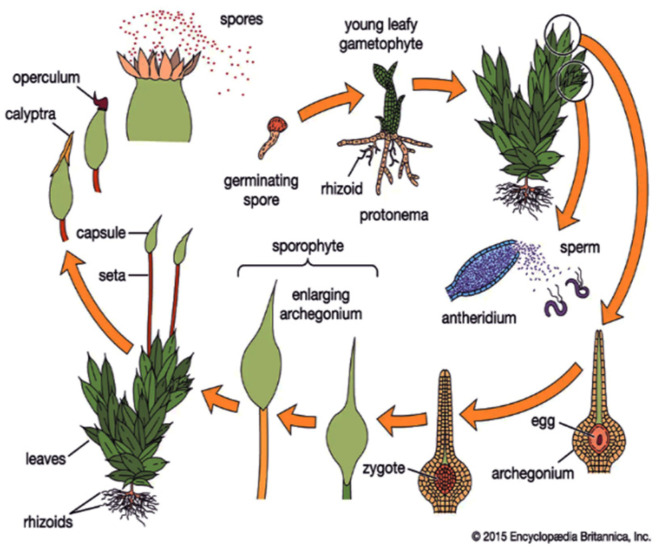
Bryophyte life cycle. Data from Encyclopedia Britannica, Inc., Chicago, IL, USA, [14].

The haploid gametophyte, a relatively simple juvenile stage of the moss, is a convenient subject for studying morphogenesis [3]. In daylight conditions, filamentous, multi-cellular moss protonemata express plagiotropic growth; in darkness, they orient negatively gravitropic. Figure 1 illustrates a detailed description of the moss life cycle.

Studies of gravitropism and gravity-dependent moss morphogenetic processes indicate the important role of gravity in plant ontogenesis. Moss spore germination, the differentiation of rhizoid and chloronemal stolons, the formation of vegetative reproductive organs, the initiation of gametophore bud development on apical protonema cells, and the formation of the sporogon are gravi-morphoses influenced by the polarizing effect of gravity [10,15,16,17,18,19,20]. Gravi-morphoses are species-specific and evolve depending on the stage of moss development and environmental factors; they represent a widespread adaptive form of growth in the life strategy of bryophytes [8,21,22]. In other systems, the dorsoventral shape of tree shoot development, gravi-dependent wood formation, the location of lateral buds in orchids and fungal fruiting bodies are well-known examples of adaptations in response to gravity [23,24,25]. Gravi-sensitive chloronemata of the species *Ceratodon purpureus* (Hedw.) Brid, *Physcomitrella patens* (Hedw.) Bruch & Schinp, (*Physcomitrium patens* (Hedw.) Mitt. and *Funaria hygrometrica* Hedw. are often used as objects in researching plant gravitropism [11,26]. In several species, moss caulonemata appear more sensitive to gravity while the gravi-response of rhizoid filaments is described as weak [15,27].

In vascular plants, within gravity-sensing cells, starch-filled amyloplasts (heavy starch particles or plastids) act as statoliths and sediment with respect to the gravity vector [28,29]. Amyloplasts are understood to be a trigger of the gravi-sensory system in the apical protonema cell. Typically, apical tip-growing cells that are gravitropic can both sense the *g*-vector and reorient their growth accordingly. Gravity vector-dependent sedimentation of amyloplasts precedes gravitropic bending of apical cells which, for example in *Ceratodon purpureus* (Hedw.) Brid., occurs 20–30 min after initiation of gravistimulation. In microgravity during spaceflight and clinorotation, amyloplasts appear evenly distributed along the entire cell and sedimentation patterns are not apparent [1,30]. Moss gravi-sensitivity manifests at the stage of gametophyte and sporophyte development as a morphogenetic adaptation to environmental influences. Many bryophytes express unique characteristics such as high tolerance to various stresses. In fact, bryophytes grow surprisingly well in diverse habitats ranging from deserts to wetlands and from tropical to polar regions. This is exemplified by the fact that the most found terrestrial plants in Antarctica are bryophytes [31,32]. DNA methylation is seen as one of the mechanisms controlling gravi-dependent protonema morphogenesis, evidenced by the increased formation of buds after gravistimulation. In the natural environment, gravi-morphoses and DNA methylation increase phenotypic plasticity of the gametophyte, an important feature for ephemeral species with a short life cycle [26,33,34].

The objective of this review is to highlight the impact of the polarizing effect of gravity on the differentiation of cells and the morphogenesis of protonemata and resulting adaptive significance at different stages of moss ontogenesis.

## 2. Gravi-Sensitivity of Bryophytes Ontogenesis

### 2.1. Gravi-Sensitivity of Sporelings

In moss, gravity serves as a trigger for the polarization of apolar spores and the subsequent development of morphologically distinct chloronema and rhizoid sporelings [35]. Species-dependent adjustment to distinct habitats and individual life strategy is influenced by their sporeling polarization and is highly variable [36]. Protonemata are the most gravi-sensitive structures in mosses.

**Figure 2:**
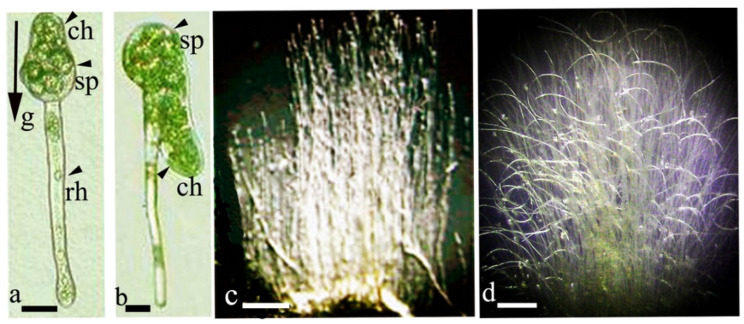
Dark-grown sporelings of *Ceratodon purpureus* and protonemata mats; (**a**)—three-day-old rhizoidal sporeling directed down (positively gravitropic) and chloronema growing upward (negatively gravitropic); (**b**)—after reorientation of the Petri dish by 360°, a chloronemal sporeling was formed and grew down parallel to the rhizoid; (**c**)—negative gravitropism of eight-day-old protonemata; (**d**)—spiral bend pattern of filaments in culture rotated for 5 days on a clinostat. Legend: sp—spore; ch—chloronema; rh—rhizoid. Scale bar: (**a**,**b**) = 30 μm; (**c**,**d**) = 7.5 mm. Data from Lobachevska et al., 2014 [15].

The development of sporelings in *F. hygrometrica* and *C. purpureus* in the dark demonstrates the strong gravitropic reaction and associated polar symmetry of growth. Chloronemata express negative gravitropism; rhizoids express positive gravitropism at a perfect 180° orientation [15,27,35]. The location of sporeling initiation occurs depending on the gravity vector. If the position of a spore culture is changed by 360° relative to the horizontal surface, a second chloronema sporeling emerges near the first rhizoidal one (Figure 2a,b). Growth reorientation to a negatively gravitropic orientation indicates the competence of cells to respond to changes relative to the gravitational vector.

The polarizing effect of gravity o” the’growth direction of sporelings is reduced in later stages of development [36]. Variability of chloronema angles in *C. purpureus* was higher (40–55°) than in *F. hygrometrica* (18–19°), potentially indicating higher gravi-sensitivity of *F. hygrometrica* sporelings. Gravity-dependent orientation of sporelings is a distinguishing feature of the moss life strategy and its morphology; in *F. hygrometrica*, sporelings may allow for faster formation of growth mats and better adaptation to changing environmental conditions.

Mosses show different growth adaptations depending on their individual life strategy (timing of the life events for best environmental conditions) [37,38]. *F. hygrometrica* has a so-called fugitive life strategy, generally surviving only for a short time (1–2 years). Funaria has a characteristically high gravi-sensitivity at the chloronema stage; in contrast, the colonist *C. purpureus* thrives where the habitat start is unpredictable, and the cultures usually last for many years. In *Ceratodon*, caulonemata are most sensitive to gravity. *C. purpureus* is a typical colonist species forming dense mats and demonstrating rapid expansion on dry, often sandy or stony substrates. In contrast, *F. hygrometrica* has a short life cycle, forms loose mats, and is adapted to wet soil conditions. In order to adapt to the local microenvironment and a short growth season, *F. hygrometrica* must establish fast and firmly anchor itself in the soil via its rhizoids. Coupled with expedited polar growth of its chloronemal sporelings, the formation of loose, but stronger mats is promoted. These morphological species-dependent differences that in part are dependent on gravity have evolved allowing for customized life strategies of these species.

### 2.2. Gravi-Sensitivity of Protonemata—The Juvenile Stage of Moss Development

The level of gravitropic sensitivity in moss protonemata appears to be species-specific and differs from species to species. For example, in *F. hygrometrica* and *C. purpureus*, sporelings and chloronemata are highly sensitive with respect to gravity [35], while gravi-sensitivity appears to be less dominant in *Dicranella heteromalla* (Hedw.) Schimp. and *Dicranella varia* (Hedw.) Schimp. In *Pohlia nutans* (Hedw.) Lindb., *D. heteromalla* and *Barbula unguiculata* Hedw., chloronema or caulonema cells only appear to respond to gravity at later stages of protonemata development [15]. In *Tortula modica* Zander (*Tortula caucasica* Broth.) and *B. caespiticium*, regenerative secondary protonemata and gametophores are gravi-sensitive; in *W. tortilis*, caulonemata are highly gravi-sensitive. Filamentous protonemata display a negatively gravitropic growth pattern in the dark (Figure 2c), while they grow plagiotropically under illumination and show spiral bend pattern filaments in culture rotated on a clinostate (Figure 2d). The unique ability of the protonema apical cell to perceive and respond to gravity made mosses a model system for the research of gravitropism in plants. Initial studies focused on *Ceratodon purpureus* [11,39], *F. hygrometrica*, *T. modica*, *P. patens*, and *Pohlia nutans* (Hedw.) Lidb. [30]. The apical protonema cell and spores of most species contain amyloplasts that sediment in response to the gravi-stimulation angle and function as statoliths [10,35,39,40]. Species-dependent differences and morphological adaptations of amyloplasts have been described [35].

### 2.3. Gravity-Dependent Development of Bud Formation

**Figure 3:**
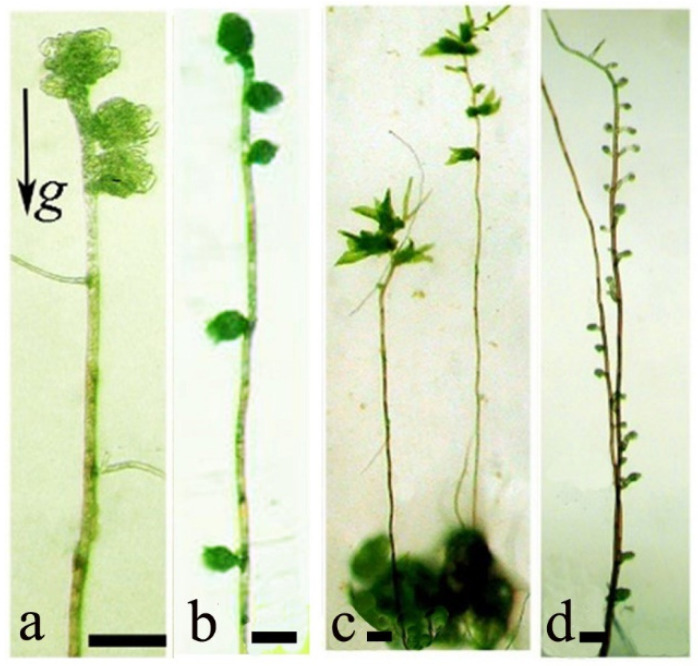
Gravitropic protonema stolons of *Tortula modica* (**a**,**b**) and *Physcomitrella patens* (**c**,**d**): (**a**)—bud development on the apical cell; (**b**)—buds form along the entire stolon under clinorotation; (**c**)—gametophores initiated from buds on the apical part of the stolon when illuminated; (**d**)—enhanced bud formation along the entire stolon after treatment by 25 µM 5-Azacytidine. Scale bar: 100 µm. Data from Lobachevska et al., 2014 [15].

The initiation and development of gametophore buds on apical cells of gravitropic protonemata was first described for *T. modica* gravitropic protonemata cultivated in nature, and later during spaceflight conditions and under clinorotation [12,36,41]. When dark-grown gravitropic protonemata was oriented vertically, phototropism was completely repressed. When reorienting these cultures horizontally under white light illumination, buds form on the apical cells (Figure 3a). Single apical cell buds developed in microgravity during spaceflight (STS–87); under clinorotation, buds initiated along the caulonema stolons (Figure 3b) are not restricted to the apical cells. When DNA methylation is inhibited by application of 5-Azacytidine (Figure 3c,d), in *P. patens*, increased bud formation is observed along most cells of the gravitropic stolon.

The formation of buds that subsequently develop into leafy shoots is a key stage in moss ontogenesis. Bud formation in apical cells of *T. modica* was described as gravity-dependent photomorphogenesis [41]. Gravity accelerates photomorphogenesis beyond the caulonema stage, obligatory for bud formation. Treatment of protonemata with cytokinin induces the formation of gametophores on caulonema stolons [18,42]. Gravity is thought to intensify the acropetal transport of cytokinin and influence bud initiation in the apical part of the stolon [16,43]. This mechanism accelerates the dominant gametophyte stage of moss ontogenesis allowing for stronger mat development, and thereby better adaptation to the environment. Clinorotation is known to randomize the effect of gravity on the cytokinins gradient; thus, gametophore buds initiate along the stolon.

### 2.4. Gravi-Sensitivity of Mosses at the Stage of Leafy Shoots and Sporogonia Formation

Typically, moss species that do not express a clear gravitropic reaction at the early chloronema stage, develop strong gravitropism in later development stages when they form gametophores and regenerative caulonemata. Leafy shoots (gametophores) of acrocarpous bryophytes are oriented negatively gravitropically. Low gravi-sensitivity of regenerative protonemata developed from shoots, compared with those developed from spores, was observed in *Bryum argenteum* Hedw. and *Dicranella varia*. However, in *B. caespiticium*, *T. truncata*. and *T. modica*, in contrast, regenerative protonemata from shoots and gametophores expressed strong gravi-sensitivity.

**Figure 4:**
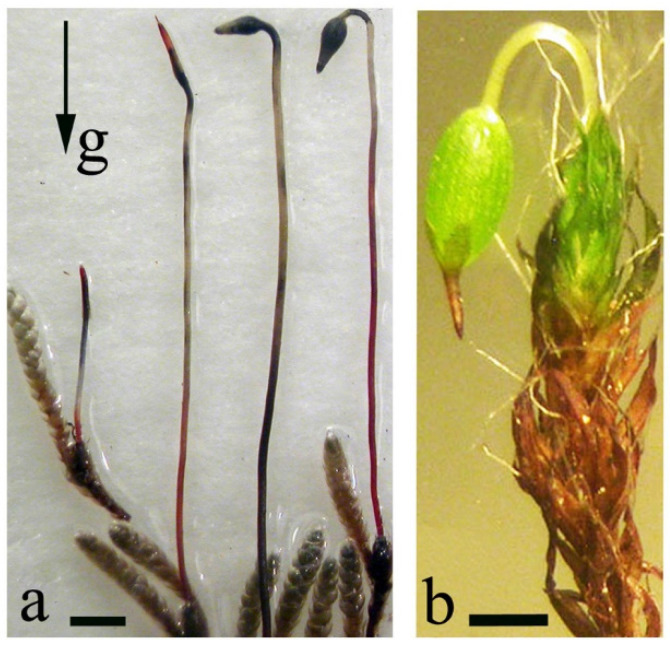
Sporogonia of *Bryum argenteum* (**a**) and *Grimmia anomala* (**b**). (**a**)—the gradual gravitropic bending of the sporogonia, (**b**)—a sporogonium with a reoriented capsule. Scale bar = 200 µm. Data from Lobachevska et al., 2014 [15].

Usually, a moss sporophyte grows negatively gravitropically at the initial stage of development and during capsule and sporogenous tissue formation; in some species, subsequently, the capsule reorients to become positively gravitropic. It was demonstrated that sporogonia form as bipolar structures with apical and basal growth centers, the direction of which changes relative to the gravity vector resulting in an inclined orientation of the capsules (Figure 4a,b). As a result of the positive gravitropic growth of the seta, the initially orthotropic orientation of the capsule inverts (at an angle of 185°) and becomes asymmetric-dorsoventral [17]. Clinorotation at the stage of the capsule formation leads to undifferentiated sporogenous tissue and/or morphological changes, e.g., spherical, often curved capsules are observed in *Bryum argenteum.* Almost vertically oriented capsules without observable bending of the seta were formed in *Pohlia nutans*. 

Gravi-dependent growth changes of the sporophyte are manifested in the reorientation of the seta and, in some cases, the shape of the capsule. Shape and spatial orientation of the capsule serves as a taxonomic feature. A positively-gravitropic orientation of the capsule ensures spore dispersion in close vicinity to the parental culture [18]. Gravi-dependent growth responses of the sporophyte during its development are seen as crucial for moss reproduction and enrichment of species diversity [17,37,38,39].

## 3. Molecular Mechanisms of Gravi-Morphoses in Bryophytes

### 3.1. The Role of Auxins in Gravitropic Response Protonemata

Controlled by auxins, the spatial orientation of the lateral branches of *C. purpureus* protonemata changes depending on the position of the cultures relative to the gravity vector. Light direction and gravitational vector-dependent branch angle distribution was observed in different moss species [19]. Growth of lateral branches and their bending angle are balanced by the action of gravity and light, and by autotropism. These factors interact at the different stages of moss ontogenesis and determine culture structure and habit under natural conditions [3,36].

When illuminated, protonemata cells form branches with branch angles of 30° in the distal part and plagiotropic growth at the stolon base; increased angles are observed under clinorotation. Auxin acts as an inducer of branch growth and controls the gravity-dependent angle and autotropism [19,44]. The mechanism of branching is based on the gradual decrease in gravi-sensitivity of the lateral branches as a result of an auxin distribution gradient with a measured increase of auxin in the apical cells of branches [18,45]. The gravitropic set-point angle (GSA) of the branches increases under treatment with 1.0 μM Indole-3-acetic acid (IAA). 1.0 μM N-1-naphthylphthalamic acid (NFA) and 20 μM Brefeldin A (PIN protein inhibitor) inhibit the IAA polar transport and the tilt angle decreases by almost 10°. The ability of organs to reorient their growth relative to the gravitational vector is based on changes in the GSA and increased morphological variability of the gametophytes [13,19]. Gravi-sensitivity of protonemata branches of the moss *C. purpureus* changed depending on the gravi-vector and gradient distribution of auxin. The suppression of polar transport involving NFA caused a decrease in anti-gravitropic offset and plagiotropic growth of lateral branches. 

### 3.2. Ca^2+^ Regulates in Gravitropism

The special importance of Ca^2+^ ions in apical cell growth has been described: the apical-basal gradient of Ca^2+^ ions is the basis of the bioelectric polarity of cells [18] and can regulate cell growth by elongation. Blocking the operation of Ca^2+^ channels and Ca^2+^-ATPase inhibits the polar flow of Ca^2+^ and gravitropic growth, and its active polar transport via operation of a Ca^2+^-ATPase pump is important for gravitropism [19]. The basipetal increase of Ca^2+^-ATPase activity was demonstrated, confirming active basipetal Ca^2+^ transport in the apical cell. Directed flow of calcium occurs constantly regardless of the cell length, i.e., calcium is not only a trigger, but a constant regulator of the cell functional activity. The ATPase activity is significantly higher on the lower side of the cell where the plastids are located. Activity increased with time and was highest 20–30 min after gravistimulation, at the time of plastid sedimentation. Amyloplast sedimentation may trigger the redistribution of Ca^2+^ ions and the related change in the polarity axis and the growth axis, thus initiating gravitropic bending. Verapamil and sodium orthovanadate are shown to inhibit the gravitropic reaction and disrupt the zonal distribution of plastids.

Work from [46] suggests that the influence of Ca^2+^ is downstream of auxin signaling and that Ca^2+^ is involved in converting auxin signals into extracellular pH changes [47]. Based on analysis of gravity-induced changes to surface pH in roots of *Arabidopsis*, it is likely that cytosolic Ca^2+^ plays a role in gravitropic signal transmission during asymmetric basipetal auxin transport along the root length. Gravitropic signal transmission is associated with asymmetric changes in cytosolic Ca^2+^ levels in the epidermis of the upper or lower side of gravi-stimulated roots [46]. Ca^2+^ concentration changes were analyzed at 0.5, 1.5, or 2 g, combined with rapid switching between hypergravity and microgravity. *Arabidopsis* seedlings possess a very rapid (sub-second timescale) gravity-sensing mechanism linearly transducing gravity intensity changes into Ca^2+^ signals [48]. The polarization of the *Ceratopteris thalictoides* (L.) Brong fern spore represents another system where Ca^2+^ fluxes have been closely linked to the gravity response. These spores exhibit a gravity-dependent Ca^2+^ current from the bottom to the top of the single cell, important in determining its early polar development [49]. Pharmacologic assays indicate endomembrane Ca^2+^-ATPases and extracellular nucleotides may play regulatory roles in the gravity response of *Ceratopteris* spores [50]. A recent study using electrophysiological analysis of Ca^2+^ currents around these spores demonstrated that hypergravity or microgravity alter transcellular Ca^2+^ currents in spores placed in the upright position [51]. These results suggest that fern spores may have similar gravity-sensing mechanisms as compared to flowering plants.

Ca^2+^ ions are necessary for the polar growth of cells as they support their polarization by controlling cytoskeleton activity. It has been shown that actin and tubulin proteins, as well as Ca^2+^ ions, are co-localized in the growth zones and sites of bud formation [19]. Microtubules were oriented parallel to the growth axis of long cell walls but not at the apex. Depolymerization of microfilaments by cytochalasin B completely inhibited apical growth [52]. Taxol, as a stabilizer of microtubules, counteracts their depolymerization, stabilizes the pool of free tubulin and, thus, affects growth processes. It was demonstrated that red light affects the polymerization of tubulin. Since tubulin immunofluorescence correlates with higher efficiency of red light in the development of mosses, it is postulated that red light controls the balance of active tubulin. Morphoses in the apical cells of *Funaria hygrometrica* at high intensity red light was accompanied by a disruption of the microtubule structure, which was simultaneously restored with the initiation of growth [52,53]. Actin microfilaments were concentrated in the apex of the cell [45] forming a labile framework that changes according to reorientation of growth. 

### 3.3. The Role of Cytoskeleton in Gravitropic Growth

Microtubules and microfilaments are not seen as the primary sensors of the gravitational stimulus but are considered part of the signaling system induced by gravity. The microtubule cytoskeleton has multiple functions in plant cells including the guidance of the intracellular motion of organelles, the targeting of enzymes involved in cell wall assembly, chromosome separation, and cell plate formation during mitosis. Microtubules in regenerating protoplasts cultured under microgravity conditions were less organized than those in the ground control, prompting further analyses of the microtubule cytoskeleton in cultures of other plant single cell types [54]. In tubulin mutants of *Arabidopsis* displaying twisted growth, hyper-gravity caused a more pronounced twisting phenotype and microtubule re-orientation was more prominent [55]. This led to the hypothesis that by influencing cellulose deposition, microtubules play an important role in the maintenance of a normal growth phenotype against the gravitational force [56]. Actin filaments, which also appear to be axially oriented along the cortex and concentrated at the apex of the tip cell, are required for tip growth. Microfilaments are closely associated with intracellular movements, such as cytoplasmic circulation, endocytosis, and exocytosis; and is related to plant response to gravity variation [18,57,58]. 

## 4. A Dominant Role of Moss Gravimorphoses in Adaptation to Extreme Conditions

### 4.1. Effects of Microgravity on Apical Cell Morphology

The apical cells of moss protonema are known to perceive and react to gravity (perception and gravitropic response in the same cell). In microgravity, amyloplasts do not sediment but are grouped closer to the apex, and sometimes small plastids were observed in the apex’s dome. The gravity force closely interfaces with internal mechanical forces involved in the sedimentation of amyloplasts [10,30,59]. After a 96-day exposure of *F. hygrometrica* to microgravity, the morphological structure of protonema cells changed. Cell walls were thinner, the number of peroxisomes increased, and the ultrastructure of organelles showed modifications, indicating premature aging of cells under these culture conditions [60]. Seventy five percent of spores that had formed during spaceflight exposure subsequently, after flight, germinated and formed normal protonemal cultures [60].

Plants correct their growth relative to light and gravity via photo- and gravi-tropic growth reactions. Gravitropic protonemata respond to unidirectional red light by phototropic bending at 1 g. The phytochrome system is known to modulate the angle of gravitropic bending in higher plants [61,62,63]. During exposure of *C. purpureus* protonemata to microgravity during the STS-87 space shuttle mission, the phototropic threshold was defined at low intensities of red light ˃140 µmol∙m^2^∙s^−1^ in microgravity [64]. Therefore, gravity affects the sensory system of photoreceptors. In low intensities of red light (λ = 640 nm, illumination intensity 0.2 μmol∙m^2^∙s^−1^), the spatial orientation of protonema lateral branches, and the angle of gravitropic bending of *C. purpureus* protonema stolons changed depending on the gravitational vector [19].

### 4.2. Spiral Growth in 1 g, Microgravity during Spaceflight, and Clinorotation

**Figure 5:**
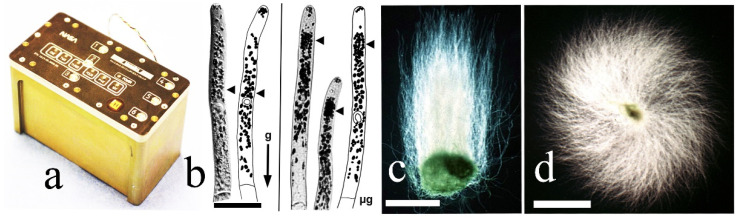
Protonemata of *Ceratodon purpureus* in dark on the ground and in space. (**a**)—BRIC-LED container for Petri dishes with moss culture; (**b**)—apical cells 7-day-old protonema stained with Lugol’s iodine solution in dark in the ground control (at left) and in space flight (at right). Arrow heads indicate chloroamyloplasts performing the statolith function; (**c**)—14-day-old protonemata growing upwards (negative gravitropism) in the ground control; (**d**)—14-day-old protonemata exhibits net clockwise spiral growth in microgravity. Bars = 40 µm (**b**), 2 mm (**c**,**d**). Data from Kern et al., 2005 [68].

Spiral growth patterns of moss cultures develop as a result of local change in the growth axis of a protonema apical cell. In light conditions, protonemata usually grow straight and form symmetrical radial cultures. Spiral growth patterns observed in nature were described for *F. hygrometrica, Fissidens bryoides* Hedw., *Barbula unguiculata*, *Pottia truncata*, *Bryum spinosum* (*Polla spinosa* (Voit) Brid. ex Loeske), *Dicranum scoparium* Hedw. and *Polytrichum* sp. [65,66,67]. Spiral growth patterns also were documented in spaceflight experiments, in which *C. purpureus* was used [11,64,68] (Figure 5).

Moss cultures were exposed to microgravity during the STS-87 mission on the NASA Space Shuttle Columbia in 1997 [64]. Moss was grown in Petri dishes that were contained in custom-designed aluminum canisters (BRIC-LED, Biological Research in Canisters-LED). Protonemata of *C. purpureus* arced clockwise in microgravity conditions during spaceflight and during clinorotation on Earth forming a spiral growth pattern [27,64,65]. Spiral growth is widespread in nature [69,70]. *B. unguiculata* forms spiral mats at 1 g when cultured in light; in contrast, *C. purpureus* grown under microgravity conditions or during clinorotation forms spiral patterns in the dark. Clinostats allow for cultures to be rotated around the horizontal axis, and, therefore, expose all parts of the culture to a continually changing position with respect to the *g*-vector; typically, at two rotations per minute. While spiral growth appears to be a gravity-dependent process, it also is controlled by light [12]. For some species, spirality represents gravity-dependent morphogenesis; for others, light-dependent morphogenesis [36].

**Figure 6:**
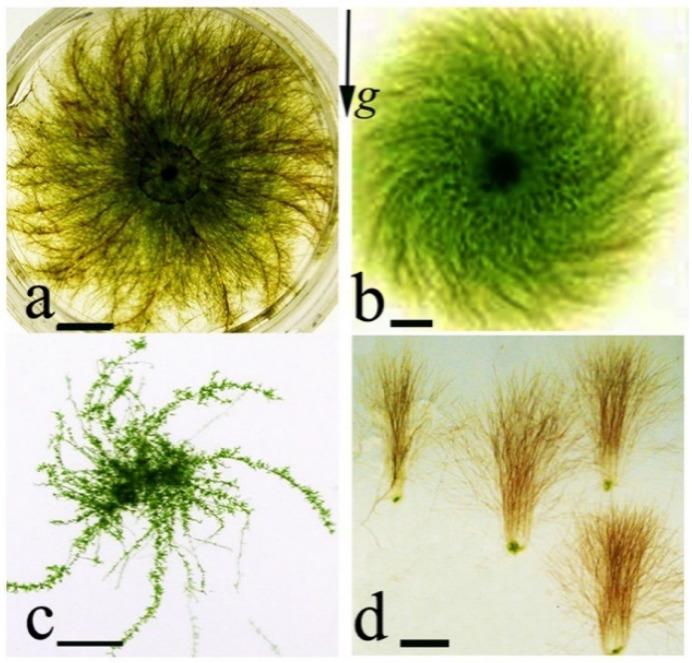
Protonema mats of different shape. (**a**)—a spiral culture of *Ceratodon purpureus* growing in the dark; (**b**)—spiral growth pattern of *Barbula unguculata* in the light; (**c**)—arc-like stolons of *Physcomitrella patens* in the light; (**d**)—cultures of *C. purpureus* during clinorotation. Scale bar: 5 mm. Data from Demkiv et al., 2006 [12].

Morphogenesis of *C. purpureus* is gravity-dependent; in other species such as *B. unguiculata* and *Ph. patens*, spiral growth patterns develop under clinorotation or in the light in horizontally placed cultures (Figure 6) [36]. During spaceflight, *C. purpureus* cultures developed spiral patterns in the absence of light and gravity, indicating that morphogenesis is controlled by endogenous factors [68]. On the cellular level, formation of an oblique cell plate in dividing apical cells and the relocation of the growth zone are the underlying mechanisms for the initiation of spiral growth patterns. The apical cells rotate around their longitudinal axis and bend despite resistance of the substrate and with disrespect of the gravity vector. Protonema morphogenesis is assumed to be regulated by endogenous factors, typically masked by gravity or light on Earth, but apparent when cultured in microgravity or during clinorotation.

### 4.3. Gravireaction of Mosses Are Influenced by Environmental Conditions

#### 4.3.1. Adaptation of *Weissia tortilis* to Arid Conditions

The gravi-response of mosses is a textbook example of how variable environmental factors, such as humidity and temperature factors, influence their development. In the arid moss species *Weissia tortilis*, Sprend, the apical cells of caulonemata growing underground and short lateral chloronema branches are sensitive to gravity [22]. In this example, the gravi-response allows the developing protonemata to emerge faster from the soil towards the light and ultimately form dense dendroid mats on the surface of the substrate (dendroids are the main stolon of the protonemata with multiple branches at their top).

**Figure 7:**
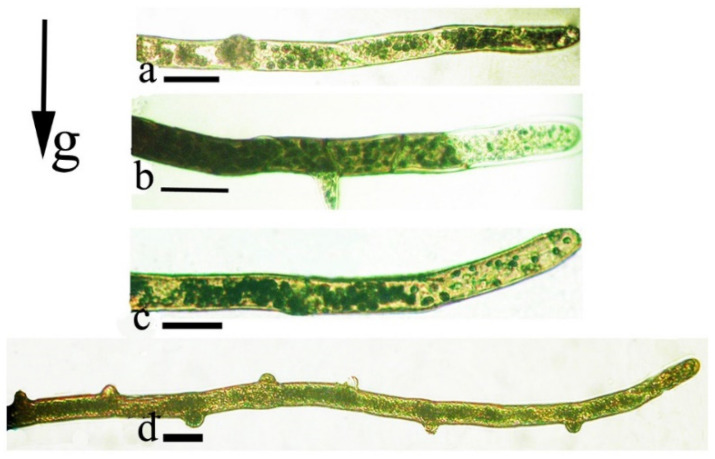
Apical cells and caulonema filaments of *Weissia tortilis*: (**a**)—amyloplasts are located in the cell apex; (**b**)—amyloplasts are concentrated in the basal zone; (**c**)—negatively gravitropic bending of an apical with apparent amyloplast sedimentation; (**d**)—branch formation during gravitropic bending of a caulonemal filament. Scale bar = 70 μm. Data from Lobachevska et al., 2021 [13].

Adaptation of mosses to microclimatic conditions influences culture shape and morphological structure [38,67,71,72]. In *Weissia tortilis*, a moss common to the arid and subarid conditions of Southern Ukraine and Central Asia, long caulonema stolons grow in the substrate surface layer and respond to the gravity vector. Above-ground positively phototropic chloronemata form on young caulonema branches. The apical cells of dark-grown *Weissia* caulonemata express unique plastid orientation (Figure 7). Caulonema filaments often branch, and those branches show strong negative gravitropism and grow upwards (Figure 7c,d).

**Figure 8:**
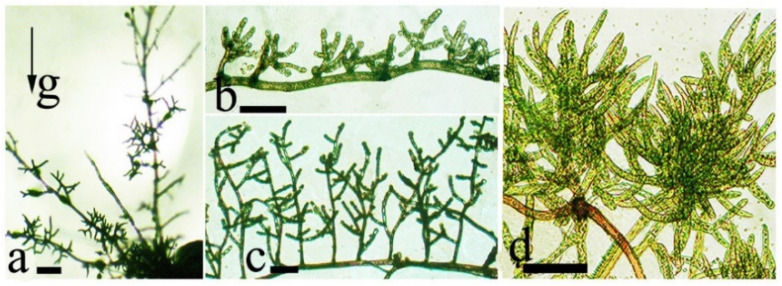
*Weissia tortilis* protonemata: (**a**–**c**)—gravi-sensitive caulonema with short chloronema dendrites; (**d**)—a culture fragment with branched dendrites. Scale bar: 120 μm. Data from Lobachevska et al., 2021 [13].

Phototropic chloronemata growing in the light form densely branched dendrites aiding a rapid culture expansion (Figure 8).

The gravi-dependent orientation of protonemata and their morphological variability are considered adaptive traits that have evolved to cope with high temperature and moisture deficiency. Formation of dendrites on negatively gravitropic stolons allows for assimilatory protonema to rapidly spread across the substrate surface, increasing the area and viability of moss protonemata. In nature, rapid culture expansion via branch formation is an important means of culture survival, ensuring access to food sources and interaction with the soil biota [22].

In natural conditions, morphological variations of protonema stolons allow for the moss to cover a larger area of distribution. Gravi-morphoses enrich the phenotypic plasticity of mosses ensuring their survival and development in extreme environments. During the seasonal water shortage in natural environments, the polarizing effect of gravity can be an active osmotic regulatory factor for vegetative reproduction and accelerated development of *Leptobryum pyriforme* (Hedw.) Wilson [20].

#### 4.3.2. Gravireaction of Moss Species from Antarctica

In the Antarctic, moss *Bryum pseudotriquetrum* (Hedw.) P.Gaertn; B.Mey & Scherb., gravi-sensitive shoots and numerous buds were formed in the leaf axils, ensuring moss reproduction during the short growing season. In ecomorphs found in the L’viv Region (Ukraine), buds also developed on rhizoids. In inclement and stressful environments such as Antarctica, gravi-responses manifesting in accelerated development of nidifugous buds in shoots aid in rapid distribution of this moss species without the need for spore formation and, therefore, ensure culture survival [73,74]. 

Apical cells of gravitropic stolons of *Bryum caespiticium* Hedw. and *Polytrichum arcticum* intensively branch in the light and develop protonemata from short chloronema stolons that then disintegrate into short fragments. Fragmentation, as a form of asexual reproduction, enables the moss plant to produce genetically identical offspring most adapted to survival in unfavorable conditions and fast colonization of large areas. In *P. arcticum* from Antarctica, only the secondary caulonema stolons are gravi-sensitive. Stolons form as a result of shoot regeneration as a strategy of rapid vegetative propagation. Chloronemal stolons, however, do not respond to gravity. Buds of gametophores and larger protonemata only develop on gravitropic protonema of *P. arcticum*, not on non-gravi-sensitive structures [74].

## 5. Conclusions

Gravity-dependent polarization of spores and the gravi-sensitivity of sporelings are the mechanisms for the young moss culture at early stages of ontogenesis to rapidly reach the substrate surface and harvest light, to firmly attach the young culture in the substrate and establish, enlarge, and ensure water supply. Gravi-morphoses, including spiral and radial protonemata growth patterns, negative gravitropic reaction in the dark, accelerated formation of gametophore buds on the apical cell and in the terminal part of the caulonemal stolon, etc. highlight the unique morphological plasticity of mosses. Resistance to extreme conditions of moisture deficit and solar irradiation of the arid moss *Weissia tortilis* is determined by the morphological structure of its protonemal mats and guided by the gravi-sensitivity of the caulonemal stage and gravitropism of chloronemal dendrites. 

Moss protonemata serve as a model system for studying the general mechanisms of the growth responses in mosses to changing environmental conditions, including gravimorphoses as well as adaptation to altered gravity in space. A species such as *Physcomitrella patens* with well-characterized genetics could be utilized as a model for documenting gravity-triggered responses and mechanisms on a molecular basis in space experiments. Moss plasticity and adaptation to microgravity conditions are important factors that eventually may aide in solving the challenges related to life support during long-duration space exploration missions, opening the possibility to utilizing mosses as components of future bioregenerative life support systems.

## Data Availability

The data that support the findings of this study are available from the authors upon reasonable request.
